# Transition from Diffusive to Superdiffusive Transport in Carbon Nanotube Networks via Nematic Order Control

**DOI:** 10.1021/acs.nanolett.3c00765

**Published:** 2023-05-10

**Authors:** Michael Wais, Filchito Renee G. Bagsican, Natsumi Komatsu, Weilu Gao, Kazunori Serita, Hironaru Murakami, Karsten Held, Iwao Kawayama, Junichiro Kono, Marco Battiato, Masayoshi Tonouchi

**Affiliations:** † Division of Physics and Applied Physics, School of Physical and Mathematical Sciences, Nanyang Technological University, Singapore 639798, Singapore; ‡ Institute for Solid State Physics, TU Wien, 1040 Vienna, Austria; § Institute of Laser Engineering, Osaka University, Suita, Osaka 565-0871, Japan; ⊥ Department of Electrical and Computer Engineering, Rice University, Houston, Texas 77005, United States; ∥ Department of Electrical and Computer Engineering, University of Utah, Salt Lake City, Utah 84112, United States; # Department of Physics and Astronomy, Rice University, Houston, Texas 77005, United States; ¶ Department of Material Science and NanoEngineering, Rice University, Houston, Texas 77005, United States

**Keywords:** carbon nanotubes, terahertz emission, photocurrent, carrier/exciton dynamics, Boltzmann equation, out-of-equilibrium modeling

## Abstract

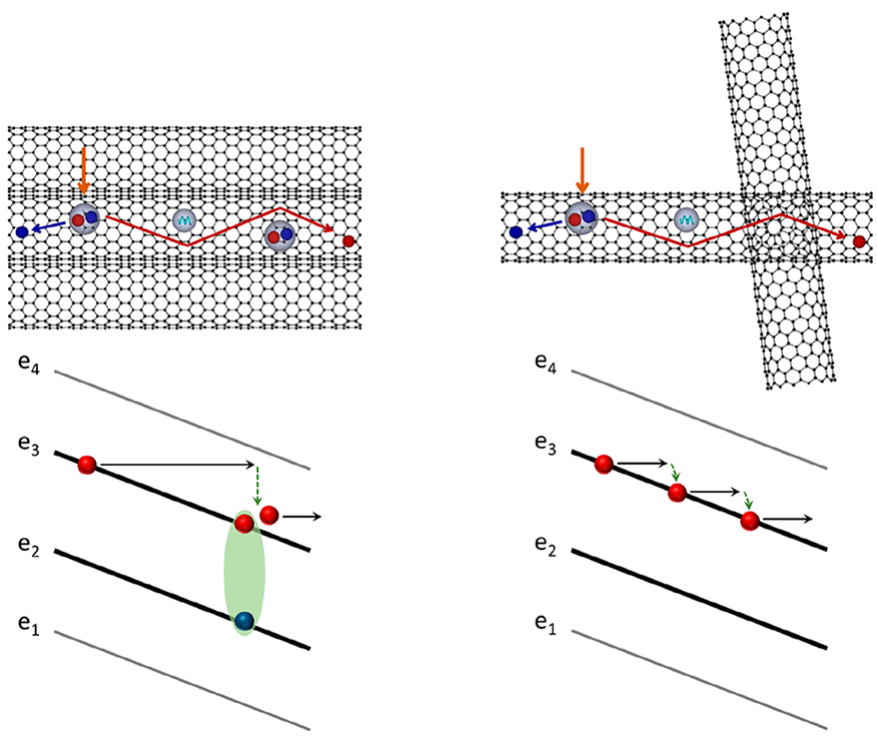

The one-dimensional confinement of quasiparticles in individual carbon nanotubes (CNTs) leads to extremely anisotropic electronic and optical properties. In a macroscopic ensemble of randomly oriented CNTs, this anisotropy disappears together with other properties that make them attractive for certain device applications. The question however remains if not only anisotropy but also other types of behaviors are suppressed by disorder. Here, we compare the dynamics of quasiparticles under strong electric fields in aligned and random CNT networks using a combination of terahertz emission and photocurrent experiments and out-of-equilibrium numerical simulations. We find that the degree of alignment strongly influences the excited quasiparticles’ dynamics, rerouting the thermalization pathways. This is, in particular, evidenced in the high-energy, high-momentum electronic population (probed through the formation of low energy excitons via exciton impact ionization) and the transport regime evolving from diffusive to superdiffusive.

The emergence of low-dimensional materials has opened up exciting possibilities for exploring new physics and functionalities inherent to their dimensionality. Carbon nanotubes (CNTs), in particular, provide an ideal platform to study the behavior of one-dimensional (1D) metals and semiconductors. Several groups have observed many complex condensed matter phenomena (Luttinger-liquid behavior,^1^ Kondo effect,^2^ Aharanov–Bohm effect,^3^ superconductivity,^4,5^ and quantum conduction^6,7^) in CNTs and also demonstrated their potential for highly efficient devices.^8,9^ Typically, the experiments are done with devices based on isolated CNTs or aligned rope-like structures (bundles) to allow access to dimension-related properties. In many applications, wide-area samples are preferable, but the 1D properties could become inaccessible if the tubes are arranged in a random fashion. Macroscopic manifestations of these 1D properties have become recently achievable^10^ due to advancements on assembly techniques that produce wafer-scale films of highly aligned CNTs.^11,12^

Despite such tremendous progress, much work is still needed to fully uncover the alignment mechanism and produce large samples of perfectly aligned, crystalline CNTs of comparable structural quality to conventional bulk single crystals. As shown in the seminal work by He et al.,^11^ the degree of alignment degrades when producing thicker films of metallic CNTs, and some chiralities of CNTs also seem more difficult to align. Other alignment methods have also been proposed,^13−17^ with different advantages and disadvantages and showing varying degrees of success. With all these difficulties, it seems logical to ask if achieving perfect alignment is really critical for the optimal performance of CNT-based devices. But, even more importantly, can alignment unlock qualitatively different dynamics that are instead suppressed in disordered CNT networks?

We attempt to answer these questions by comparing the dynamics of photogenerated quasiparticles under the influence of external electric field in aligned and random CNT networks. Specifically, we employ a combination of experiments (terahertz (THz) emission and photocurrent measurements) and numerical simulations to understand the relevant scattering processes in CNTs. The experimental techniques allow us to access the ultrafast current (within picosecond time scales) leading to THz radiation and the slow current components through the total photocurrent. Combined with the numerical simulations that accurately model out-of-equilibrium processes,^18^ we have applied these techniques to unravel the roles of spontaneous exciton dissociation and exciton impact excitation in THz and photocurrent generation, respectively.

Furthermore, we show that electrons undergo superdiffusive (diffusive) motion in aligned (random) CNT networks, depending on the number of scatterings with phonons and impurities. In the diffusive regime, momentum-randomizing scatterings are frequent, and the bias-induced acceleration only perturbatively alters the electronic population. Conversely, when momentum-randomizing scatterings become rarer, the asymmetric displacement of the electronic population within the Brillouin zone becomes sizable (leading to the so-called superdiffusive regime) and can even trigger new thermalization pathways that were previously forbidden due to energy or momentum thresholds. In the case of CNTs, we will show that this transport behavior directly affects the kinetic energy gain of electrons by acceleration in the electric field and the possibility of producing additional excitons by impact generation.

We fabricated dipole-type photoconductive antenna (PCA) structures (Figure 1a) on top of the CNT films using standard photolithography techniques (see Supporting Information for details of device fabrication and experimental setup). We prepared three CNT-based PCA devices that represent possible configurations: aligned CNT films with the tube axis direction parallel (Sample 1) and at 45° with respect to the electric field (Sample 2) and a randomly oriented film (Sample 3), as illustrated in Figures 2a, 2b, and 2c, respectively. The CNT films had similar chirality purities (Figure 1b) but with significantly different degrees of alignment. Specifically, based on the calculated values of the reduced linear dichroism (LD^r^),^11^ the aligned CNT samples had about two times better alignment compared with the random film (inset, Figure 1b). The nematic order parameter (*S*) of Samples 1 and 2 was ∼0.38; note that *S* = 0 for a completely random distribution and *S* = 1 for a perfectly aligned film.^18,19^

**Figure 1:**
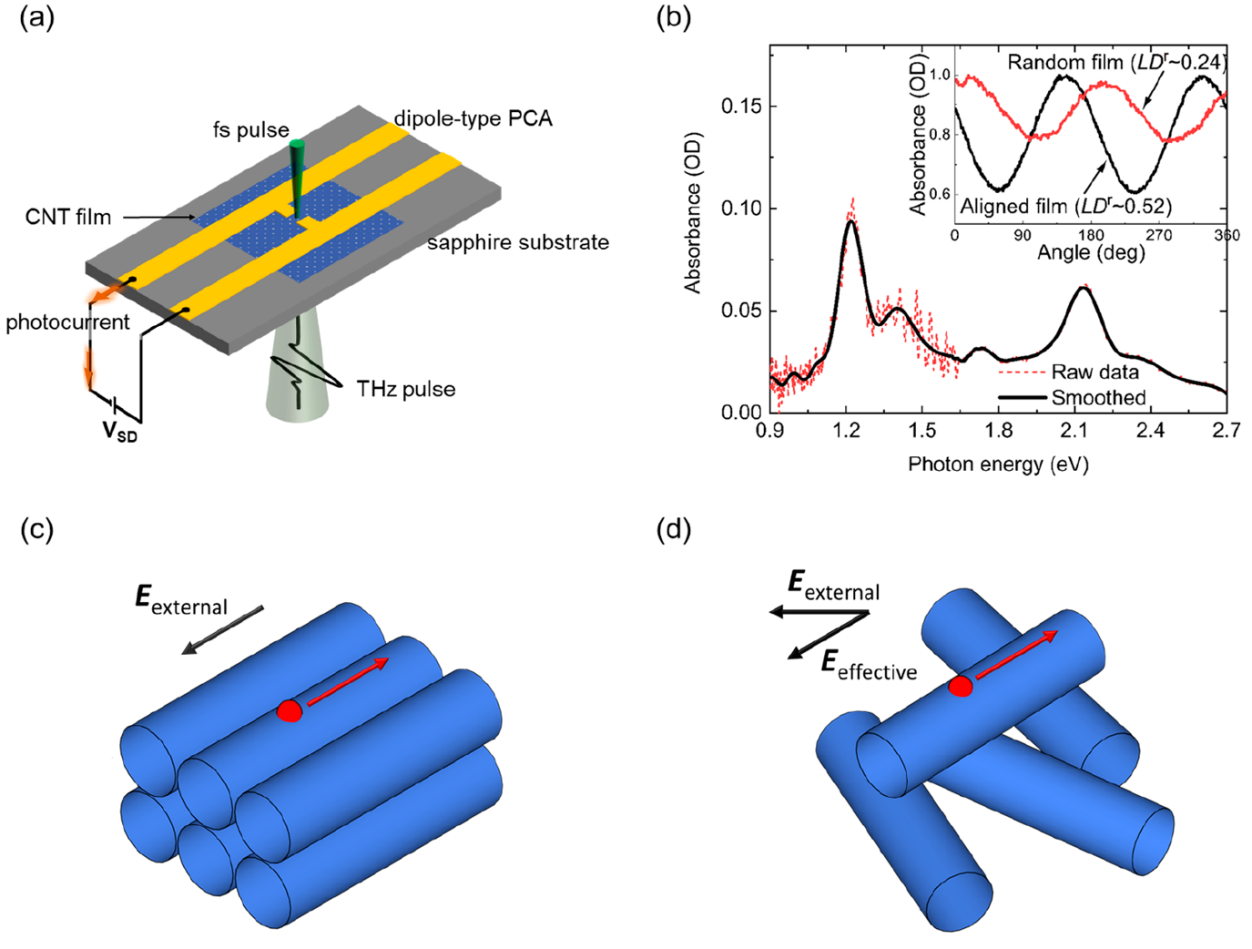
(a) Device schematic diagram. (b) Unpolarized light absorption and polarization-dependent absorption at 660 nm (inset) of (6,5) CNT films. (c) Fully aligned CNT film with electric field parallel to the alignment direction. (d) Completely random CNT film showing a reduced effective electric field along the tube.

**Figure 2:**
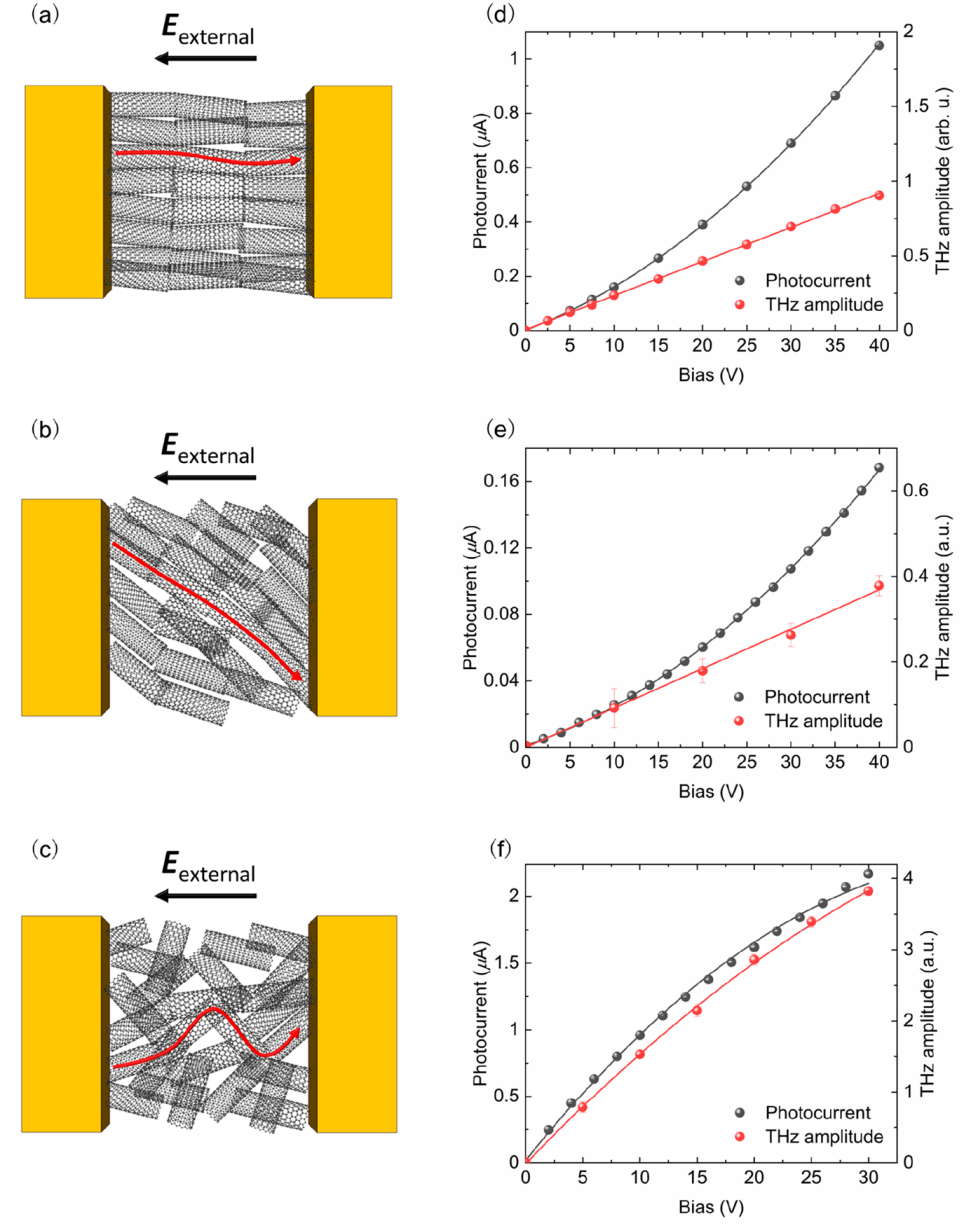
Probable carrier conduction path and bias dependence of THz amplitude and photocurrent for CNTs aligned parallel (a and d) and tilted relative to the electric field (b and e) and random CNTs (c and f), respectively.

The device configuration (i.e., degree of alignment and direction relative to the external electric field) affects the carrier transport direction in the CNT network. First, the effective bias voltage across each individual tube depends on its orientation with respect to the electric field (Figures 1c and 1d, also see Supporting Information). Second, the electrical conductivity in the CNT film is highly anisotropic,^20,21^ with the ratio of conductivities between the parallel and perpendicular directions as high as 60.^11^ In a CNT network composed of interconnected nanotubes, the carriers will follow a path with the least electrical resistance, which is nominally determined by tube chirality, density, orientation, and connectivity.^22−25^ In our devices, the red lines indicated in Figures 2a–2c depict one probable path of carriers between the electrodes based on the degree of alignment and overall tube orientation relative to the electric field.

Aside from the differences in the propagation path of carriers in the samples, the bias dependence of THz emission and photocurrent have different behaviors as shown in Figures 2d–2f. We observed a superlinear increase in photocurrent with bias and a linear increase in the THz amplitude with bias in both aligned samples (Samples 1 and 2) (Figures 2d and 2e), whereas in Sample 3 (random CNT), both THz amplitude and photocurrent show a saturation-like sublinear behavior with bias (Figure 2f). To explain these differences, we employed a newly developed numerical approach to the Boltzmann transport and scattering equation^18,26,27^ to understand the mechanisms that contribute to THz and photocurrent generation in CNTs, the influence of the external electric field on the dynamics of photogenerated quasiparticles, and the effect of the degree of alignment and direction with respect to the electric field.

We considered two extreme cases in the current numerical simulations: a fully aligned CNT network with an electric field applied parallel to the alignment direction (Figure 1c) and a completely random network of CNTs (Figure 1d). Identical numerical parameters were implemented in the two cases, but we increased the electron–phonon (acoustic) scattering amplitude by ten times in the random CNT network to emulate a sharp increase in impurity-like scattering due to the multiple tube distortions at the tube intersections. Additionally, we account for the angle formed between the electric field and the CNTs in the random case (see Supporting Information for details).

The schematic diagram in Figure 3a describes the processes occurring at different time scales for aligned and random CNTs, similar to what we have proposed previously.^18^ In our simulations, we assume that the laser excitation only generates *E*_22_ excitons and not free electron–hole pairs due to large differences in oscillator strengths^28^ and also because our polarization- and excitation energy-dependence data indicate that both THz and photocurrent generation are initially triggered by *E*_22_ exciton generation (i.e., maximum responses occur for light linearly polarized parallel to the tube axis and at around 2.14 eV excitation, see Figures S2 and S3 in the Supporting Information). The *E*_22_ excitons then partially decay through phonon scatterings^29,30^ into bright and dark *E*_11_ excitons with similar energies but larger momenta.

**Figure 3:**
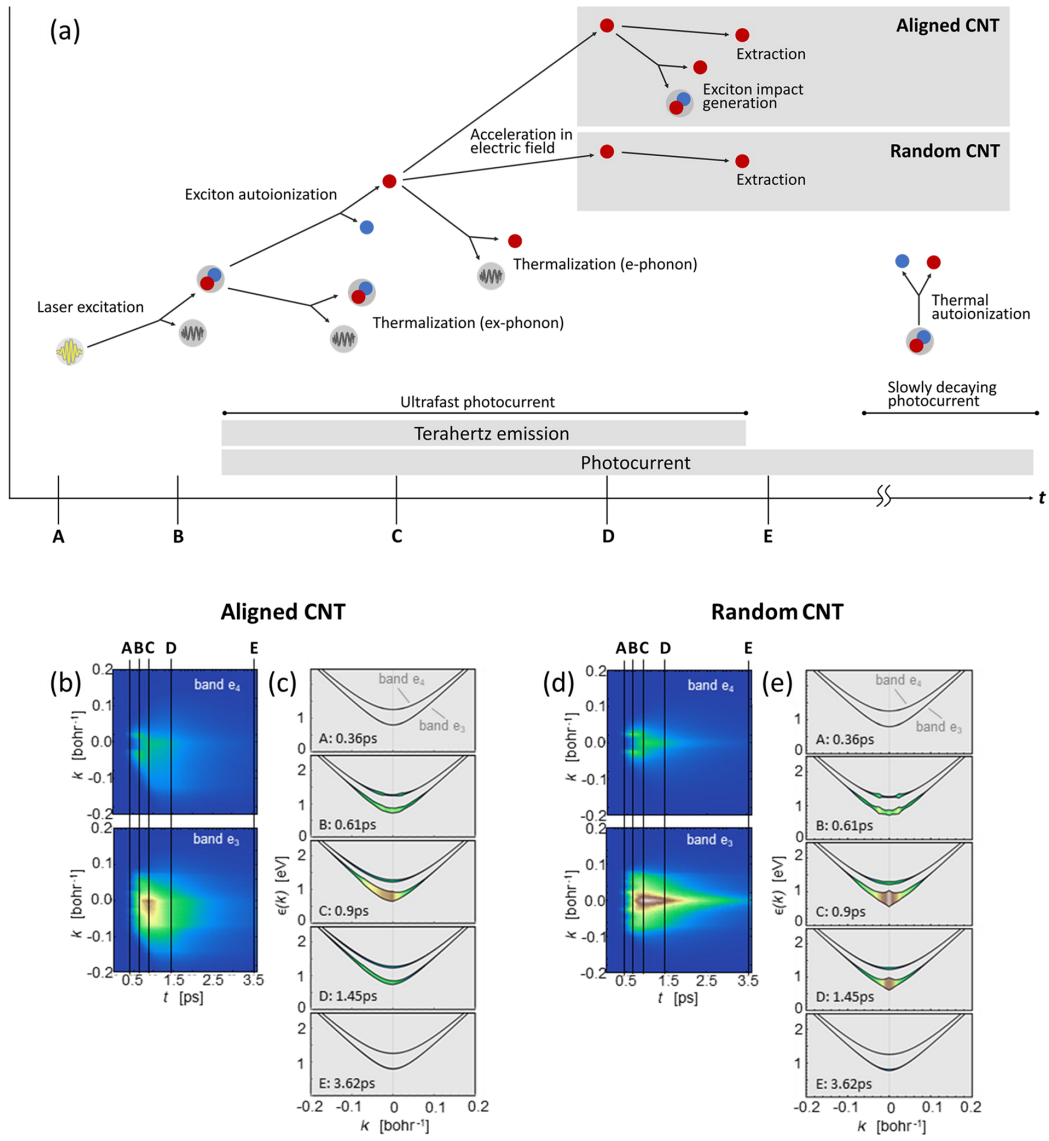
Qualitative description of the most relevant scattering process at different time scales in aligned and random CNTs (a), with the corresponding band-resolved density plots for the electronic bands (b and d) and time snapshots of the populations (c and e), respectively.

These highly energetic excitons undergo further scatterings with phonons until they reach the bottom of their respective excitonic bands. Being charge-neutral, the excitons are not accelerated by the electric field and are more likely to survive longer (at least longer than the characteristic extraction time of 1 ps for carriers) before being annihilated through radiative or nonradiative recombination (this process is not included in the simulations). During this time, the high-energy tail of these excitons will slowly autoionize into additional free carriers due to the high temperature (Figure 3a). The measured total photocurrent is the sum of this slow (i.e., low-frequency) current (which does not lead to THz emission) and the ultrafast current that generates the THz emission.

The origin of this ultrafast current, on the other hand, is closely related to the initial *E*_22_ excitons generated by laser excitation.^18^ A fraction of these excitons autoionize into free electrons (holes) in the conduction (valence) bands through spontaneous dissociation,^31^ and their acceleration in the electric field and the subsequent extraction (turning-off) produce the impulsive current responsible for the THz emission (*E⃗*_THz_ ∝ ∂*J⃗*/*∂t*) (Figures 4a and 4b). The same autoionization scattering amplitudes were used for both aligned and random CNTs. This means that the initial population of free carriers will be proportional to the number of absorbed photons and that the THz emission amplitude should linearly depend on the pump power, which we confirmed experimentally (see Figure S4 in the Supporting Information).

**Figure 4:**
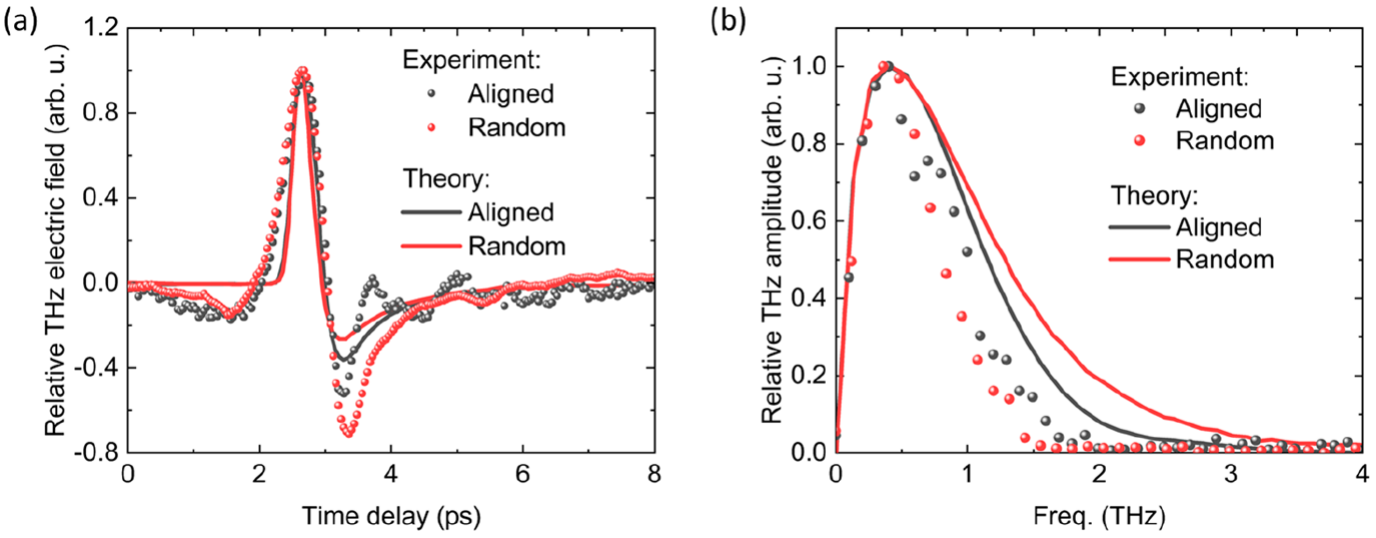
Theoretical and experimental THz emission for aligned and random CNTs in the (a) time and (b) frequency domains.

One of the effects of randomness in the random CNT network is an increase in impurity-like scatterings arising from multiple tube distortions coming from the intersecting tubes (Figure 1d). This leads to very different time evolution of the electronic band populations between aligned (Figures 3b and 3c) and random (Figures 3d and 3e) CNT networks. After the partial autoionization of *E*_22_ excitons (*B* in Figures 3b–3e), the free carriers are accelerated by the electric field, which leads to an asymmetry between positive and negative momenta in the electronic population. This acceleration is limited by momentum-randomizing scatterings (electron–phonon and electron–defect)^32,33^ that prevent indefinite gain of kinetic energy. While electron–phonon scatterings are expected to maintain approximately the same strength in both samples, the scatterings with lattice defects are amplified in random CNTs, leading to a diffusive-like (close to dynamic equilibrium) carrier transport (*C* and *D* in Figures 3d and 3e), while we see a behavior closer to ballistic carrier transport (and a very asymmetric distribution of electrons in *k*-space) for aligned CNTs (*C* and *D* in Figures 3b and 3c).

The electron transport behavior (diffusive or superdiffusive) within the first few picoseconds has an important influence on the final number of excitons in the system and to the photocurrent through the slow thermal autoionization of excitons surviving in the end (Figure 3a). Exciton impact generation, wherein an electron loses energy by scattering with an impurity and generates a low-energy exciton in the process, becomes important for electrons having an energy above a certain threshold.^18^ Due to energy conservation, this four-leg scattering channel (see Supporting Information for details) becomes active only for electrons with an energy larger than 1.2 eV above the band bottom. In random CNTs, the electrons’ momentum is frequently randomized due to the increased impurity scatterings, preventing the carriers from reaching large momenta and therefore large energies, whereas the superdiffusive transport in aligned CNTs results in a significant number of electrons gaining sufficient energy to undergo exciton impact generation. The impact generation becomes favorable at high bias due to the higher energy gain of electrons. Finally, the photocurrent in aligned CNTs is expected to show a superlinear dependence on bias (Figure 5b) as a consequence of exciton impact generation (and the subsequent slow thermal autoionization), but a linear behavior is expected in the case of random CNTs (Figure 5a).

**Figure 5:**
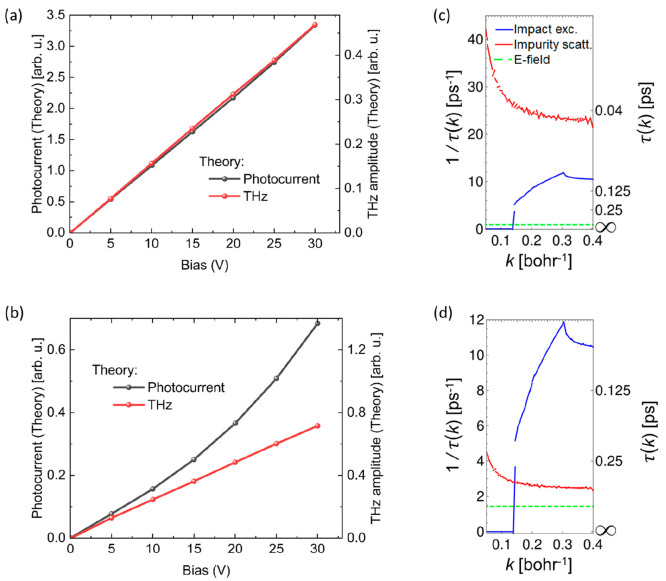
Theoretical bias dependence of THz amplitude and photocurrent for (a) random and (b) aligned CNTs. Calculated momentum-resolved scattering rates for electrons in the e_3_ band for (c) random and (d) aligned CNTs. The electric field time scale is computed as the time it takes to an electron to be accelerated ballistically to the exciton impact generation threshold in the case of bias 30 V.

For a closer analysis of the diffusive regime, we need to estimate how large is the asymmetric high-momentum tail of the electronic distribution at the energy threshold for exciton impact generation. To do this we compare the typical time it takes for electrons to be accelerated beyond the energy threshold (green dashed line in Figures 5c and 5d) and the momentum-randomizing total electron–phonon and electron–impurity scattering lifetime (red line). When the latter is shorter, electrons, on average, will not have time to be accelerated to the energy threshold, as they will be scattered and have their momentum randomized. This is the so-called diffusive transport regime, and it leads to small high energy populations. On the other hand, when scattering lifetimes are longer, the electrons will have enough time to build up momentum under the bias and climb to higher energies. If the scattering time was infinitely long, the electrons would undergo a ballistic acceleration. In the case of aligned CNTs the scattering time is not infinity but comparable to the time it takes the carriers to accelerate to above the threshold: the motion is said to be superdiffusive, and a sizable population will reach the threshold.

Notice that yet another time scale is relevant: the momentum-resolved exciton impact ionization lifetime (blue line in Figures 5c and 5d). If this is not sufficiently short, the vast majority of the electrons above the threshold will decay through electron–phonon or electron–impurity scatterings, making our probing technique inefficient. Therefore, we also compare this third time scale to understand the fraction of the electrons above the threshold that actually creates low energy excitons through impact generation. Indeed we find that in the case of random CNTs (Figure 5c) electron–defect scattering lifetimes are considerably shorter than the time it takes to reach the threshold energy, leading to the standard diffusive regime. The situation is importantly different in the aligned case (Figure 5d), where the momentum-relaxing scattering lifetimes are around half the acceleration times, a signature of a superdiffusive regime.

The dynamics presented above explain well the properties of THz emission and photocurrent in our CNT-based devices. The ultrafast current contributing to THz emission linearly depends on the number of absorbed photons (Supporting Information) and the strength of the electric field (Figures 2d and 2e). On the other hand, the photocurrent has an additional slow component produced by thermal autoionization of excitons, and the final number of these excitons is affected by bias-driven impact generation. This leads to superlinear photocurrent with bias, which we see in Figures 2d and 2e for Samples 1 and 2 (aligned CNTs), respectively. However, for Sample 2, the deviation from linearity of the photocurrent occurs at a higher applied bias (Figure 2e). This is due to the fact that at the same applied bias the effective electric field across the tubes in Sample 2 is smaller compared to Sample 1 because of the mismatch between the tube orientation and the field. The superlinearity completely disappears in Sample 3 (random CNTs) because of the suppression of exciton impact generation, and instead we observe a saturation-like behavior in both THz amplitude and photocurrent versus bias (Figure 2f). No saturation effects were included in the modeling, which explains the discrepancy between experiment (Figure 2f) and theory (Figure 5a). This kind of saturation in CNTs could result from field-induced saturation effects,^34^ reduced mobility by optical phonon emission at high fields,^35^ or ionized-impurity-like scattering due to accumulated charges in CNT intersects,^36^ none of which were incorporated in the numerical simulations and are beyond the scope of the present study.

We have shown how the initial electron transport in a CNT network is affected by the degree of alignment and direction relative to the applied electric field. This transport behavior (superdiffusive or diffusive) strongly affects the kinetic energy gain of electrons by acceleration in the electric field and the production of additional excitons by impact generation and reroutes thermalization pathways.

These findings have great implications for the design of CNT-based optoelectronic devices. Devices that do not operate at high frequencies, such as photodetectors, could potentially benefit from the slow thermal autoionization of impact-generated excitons for increased responsivities. For such devices, highly aligned CNTs can be incorporated in narrow structures designed to generate high electric fields to enhance exciton impact generation while minimizing the required applied voltage. On the other hand, devices like THz emitters could benefit more from increased photon absorption to generate more charge carriers (ultrafast current) by spontaneous exciton dissociation. Without considering electron mobility and coherence effects, perhaps unaligned but thicker CNT films (which are easier to produce) are better for THz applications. Similar considerations might be viable for other low-dimensional materials where the dynamics of carriers and excitons are affected by device and material geometries.

Last but not least, we have also demonstrated the potential of THz and photocurrent measurements together with our newly developed numerical simulations in elucidating momentum-dependent ultrafast dynamics in CNTs under strong electric fields. Time-resolved pump–probe experiments are typically employed to study the ultrafast dynamics of carriers, excitons, and phonons in CNTs, but these measurements are usually limited to zero or low electric field conditions due to difficulties in probing the region with a high electric field that are usually in the micron scale. By combining THz and photocurrent experiments, we are able to access charge carrier creation at distinct time scales after photoexcitation, thereby allowing us to create an accurate microscopic model of the dynamics.

## Supporting Information Available

Additional information related to the device fabrication, experimental setup, numerical simulations, and other supporting data (PDF)
