# Supplementary material for: Transition from Diffusive to Superdiffusive Transport in Carbon Nanotube Networks via Nematic Order Control
Source: Nano Lett. 2023 May 10;23(10):4448–55. doi: 10.1021/acs.nanolett.3c00765 (PMC10214483; doi:10.1021/acs.nanolett.3c00765)
Supplement: Supplementary file 1 — nl3c00765_si_001.pdf [file nl3c00765_si_001.pdf]

Supplementary Information:

# Transition from Diffusive to Superdiffusive Transport in Carbon Nanotube Networks via Nematic Order Control

Michael Wais*[,a,b], Filchito Renee G. Bagsican*[,c,d], Natsumi Komatsu[e,f], Weilu Gao[g], Kazunori Serita[c], Hironaru Murakami[c], Karsten Held[b], Iwao Kawayama[c,h], Junichiro Kono [e,i,j,a,c,] , Marco Battiato[a] and Masayoshi Tonouchi[c]

a. Division of Physics and Applied Physics, School of Physical and Mathematical Sciences, Nanyang Technological University, Singapore
b. Institute for Solid State Physics, TU Wien, 1040 Vienna, Austria
c. Institute of Laser Engineering, Osaka University, Suita, Osaka 565-0871, Japan
d. *Current address*: Femtosecond Spectroscopy Unit, Okinawa Institute of Science and Technology Graduate University, Onna-son, Okinawa 904-0495, Japan
e. Department of Electrical and Computer Engineering, Rice University, Houston, Texas 77005, United States
f. *Current address*: Department of Chemical and Biomolecular Engineering, University of California, Berkeley, Berkeley, CA 94720, United States
g. Department of Electrical and Computer Engineering, University of Utah, Salt Lake City, Utah 84112, United States
h. *Current address*: Graduate School of Energy Science, Kyoto University, Kyoto 606-8501, Japan
i. Department of Physics and Astronomy, Rice University, Houston, Texas 77005, United States
j. Department of Material Science and NanoEngineering, Rice University, Houston, Texas 77005, United States

*These authors contributed equally to this work.

Corresponding authors: **tonouchi.masayoshi.ile@osaka-u.ac.jp, marco.battiato@ntu.edu.sg, filchitorenee.bagsican@oist.jp**

# 1. Materials and methods

## 1.1. Device fabrication

We prepared the CNT samples used in this work using the controlled vacuum filtration (CVF) method[1,2], which produces wafer-scale monodomain films of highly aligned and dense CNTs. Briefly, we passed a low concentration of CNTs dispersed in water with a surfactant (sodium deoxycholate, Sigma Aldrich) through a polycarbonate filter membrane (Nuclepore Track-Etched (Hydrophilic) Membranes, GE Healthcare Life Sciences) under a controlled pressure gradient. We then deposited the resulting CNT films on sapphire substrates prior to electrode deposition by a wet transfer method. We determined the CNT alignment direction relative to the substrate using polarization-dependent absorbance measurement[1,3], as well as via optical inspection of the micro-groove direction in the filter membrane[2]. We used purified CNTs in conjunction with the CVF method to produce highly aligned and chirality-enriched (6,5) semiconducting CNT films.

The dipole-type photoconductive antenna (PCA) structures were fabricated on top of the CNT films using standard photolithography techniques. Using a combination of photoresists (LoR5B and S1818G, MicroChem), we produced an undercut structure to facilitate easier lift-off of excess metal. We initially deposited a 3-nm layer of titanium as an adhesion layer, which was followed by a thicker layer of gold (200 nm). Finally, the PCA structure was obtained by careful removal of the excess metal using an organic solvent stripper (Remover PG, MicroChem) to minimize damage to the CNT film. We bonded very thin copper wires on opposite sides of the metal strip to supply the bias across the CNT films during experiments.

## 1.2. THz emission and photocurrent setup

We fed femtosecond-duration pulses of near-infrared radiation from a Ti:sapphire laser (MaiTai HP, Spectra Physics) into an optical parametric oscillator (OPO) system (Inspire HF100, Spectra Physics) to produce a widely tunable optical beam (490-750 nm /1.65-2.53 eV) capable of exciting the (6,5) CNTs around the $E_{22}$ resonance peak (580 nm, 2.14 eV). We used the image formed by reflection of the excitation (probe) beam to locate the active dipole region of the PCA structure in the sample (detector). The emitted THz radiation (in transmission configuration) was first collimated by a hemispherical MgO lens (~5 mm diameter) directly attached to the backside of the sample substrate and then guided by a pair of off-axis parabolic mirrors into the LT-GaAs detector. The detector

was gated by the time-delayed fundamental beam (undepleted, 820 nm) from the OPO for time-domain detection. We measured the generated photocurrent in the sample using the same contacts that apply the bias. Both THz and photocurrent were measured by lock-in detection. All experiments were done at room temperature with the samples kept under vacuum conditions ($1 \times 10^{-3}$ Pa) inside a cryostat chamber with quartz windows for optical access.

### 1.3. Numerical simulations

We employed a newly developed numerical approach to the Boltzmann transport and scattering equation to understand the microscopic processes involved in THz radiation and photocurrent generation in the CNT-based devices[4–6]. Similar to our previous work[4], we explicitly treated four electron bands ($e_{1...4}$), three phonon/defect bands (acoustic phonon, optical phonon, impurity/defect), three exciton bands (dark $E_{11}$, bright $E_{11}$ and bright $E_{22}$) and a photon band. Realistic dispersions were adopted for electrons, excitons, and photons and an effective phonon/defect band for the large number of lattice modes and imperfections. We used the same 52 scattering channels as in our previous work[4], but with some modifications in the scattering amplitudes (see Theoretical Aspects in section 2 of this Supporting Information file for more details) which we found were necessary to better fit both the present experimental data and those in [4] as shown in Fig. S1. One of the most notable changes implemented is the shorter characteristic time ($\tau_{\text{extr.}} \approx 1$ ps) to model the extraction of free carriers in the system. This means that in our current model, instead of physical extraction into the metallic leads that are 6 μm apart (corresponding to $\tau_{\text{extr.}} \approx 5$ ps), we assume that recombination of carriers at the end of the CNT length ($l_{\text{CNT}} \approx 420$ nm) is the main decay mechanism responsible for switching-off of the electric current. This choice affects the shape of the time-domain THz waveform ($\vec{E}_{\text{THz}} \propto \partial \vec{J} / \partial t$), with a faster switching-off mechanism leading to a sharper negative peak after the main positive one. Notice that this leads to a clear improvement in the agreement between experiments and theory in Ref. [4] as well. This treatment is also in line with recent reports indicating that the ends/edges in CNTs[7,8] and other low-dimensional materials[9] act as active sites for recombination.

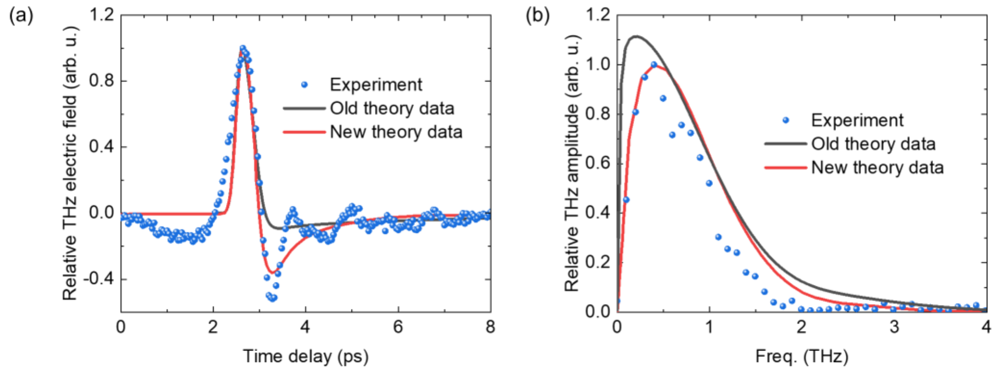


Fig. S1. Comparison of experiment with the old[4] and new theoretical THz emission in (a) time and (b) frequency domains. The faster switching-off mechanism leads a better agreement between experiment and theory.

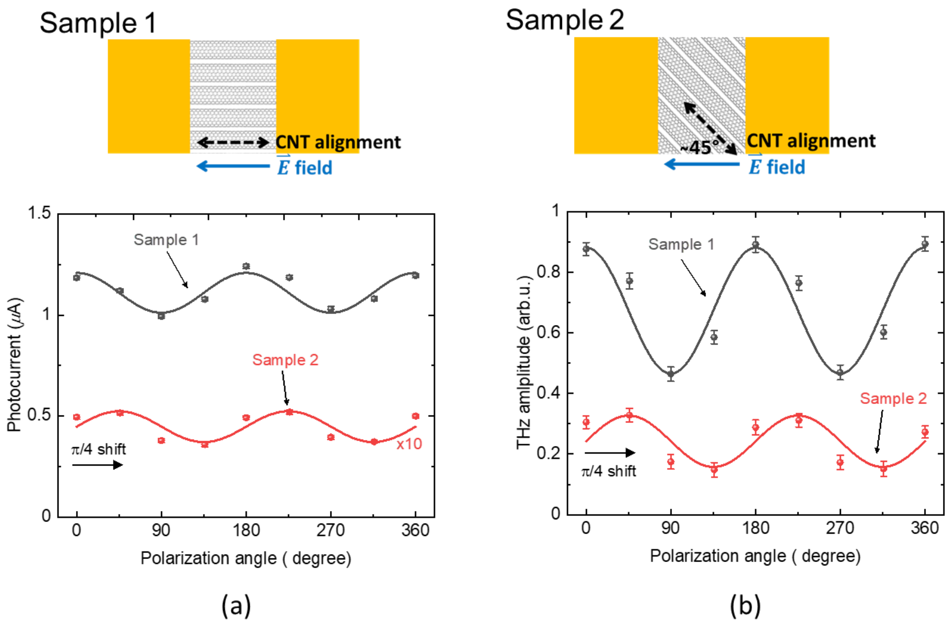


Fig. S2. Polarization-dependence of photocurrent (a) and THz amplitude (b) for samples aligned parallel (Sample 1) and at 45° (Sample 2) with respect to the electric field.

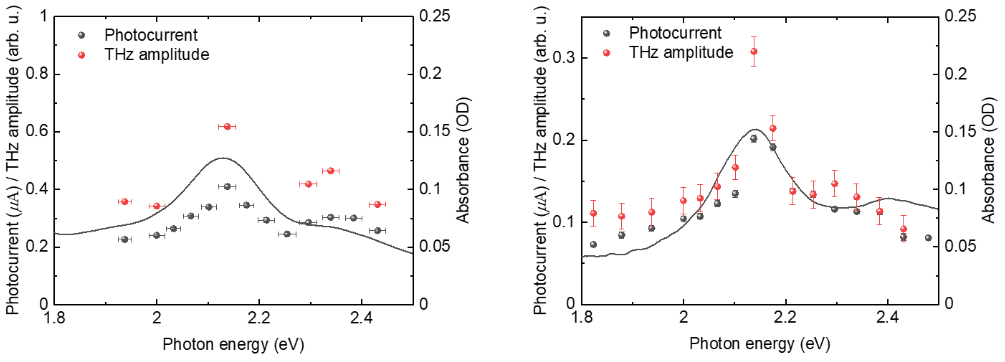


(a) (b)

Fig. S3. Photon energy-dependence of THz emission amplitude and photocurrent for random (a) and aligned (b) CNTs.

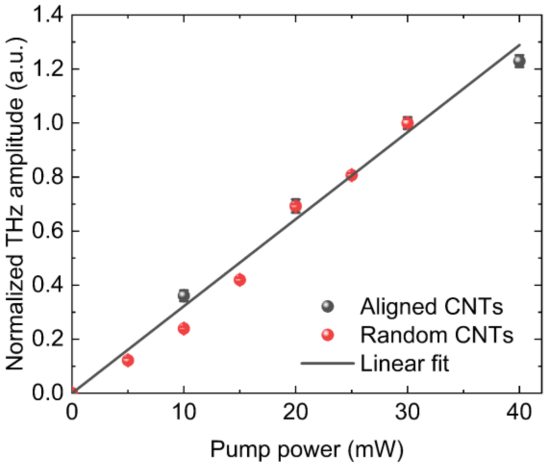


Fig. S4. Dependence of THz emission amplitude on excitation power.

## References:


(1) He, X.; Gao, W.; Xie, L.; Li, B.; Zhang, Q.; Lei, S.; Robinson, J. M.; Hároz, E. H.; Doorn, S. K.; Wang, W.; Vajtai, R.; Ajayan, P. M.; Adams, W. W.; Hauge, R. H.; Kono, J. Wafer-Scale Monodomain Films of Spontaneously Aligned Single-Walled Carbon Nanotubes. *Nat Nanotechnol* **2016**, *11* (7), 633–638. https://doi.org/10.1038/nnano.2016.44.

(2) Komatsu, N.; Nakamura, M.; Ghosh, S.; Kim, D.; Chen, H.; Katagiri, A.; Yomogida, Y.; Gao, W.; Yanagi, K.; Kono, J. Groove-Assisted Global

Spontaneous Alignment of Carbon Nanotubes in Vacuum Filtration. *Nano Lett* **2020**, *20* (4), 2332–2338. https://doi.org/10.1021/acs.nanolett.9b04764.

(3) Katsutani, F.; Gao, W.; Li, X.; Ichinose, Y.; Yomogida, Y.; Yanagi, K.; Kono, J. Direct Observation of Cross-Polarized Excitons in Aligned Single-Chirality Single-Wall Carbon Nanotubes. *Phys Rev B* **2019**, *99*, 035426. https://doi.org/10.1103/PhysRevB.99.035426.

(4) Bagsican, F. R. G.; Wais, M.; Komatsu, N.; Gao, W.; Weber, L. W.; Serita, K.; Murakami, H.; Held, K.; Hegmann, F. A.; Tonouchi, M.; Kono, J.; Kawayama, I.; Battiato, M. Terahertz Excitonics in Carbon Nanotubes: Exciton Autoionization and Multiplication. *Nano Lett* **2020**, *20* (5), 3098–3105. https://doi.org/10.1021/acs.nanolett.9b05082.

(5) Wais, M.; Held, K.; Battiato, M. Numerical Solver for the Time-Dependent Far-from-Equilibrium Boltzmann Equation. *Comput Phys Commun* **2021**, *264*, 107877. https://doi.org/10.1016/J.CPC.2021.107877.

(6) Wadgaonkar, I.; Jain, R.; Battiato, M. Numerical Scheme for the Far-out-of-Equilibrium Time-Dependent Boltzmann Collision Operator: 1D Second-Degree Momentum Discretisation and Adaptive Time Stepping. *Comput Phys Commun* **2021**, *263*, 107863. https://doi.org/10.1016/J.CPC.2021.107863.

(7) Rajan, A.; Strano, M. S.; Heller, D. A.; Hertel, T.; Schulten, K. Length-Dependent Optical Effects in Single Walled Carbon Nanotubes. *Journal of Physical Chemistry B* **2008**, *112* (19), 6211–6213. https://doi.org/10.1021/jp077144l.

(8) Heller, D. A.; Mayrhofer, R. M.; Baik, S.; Grinkova, Y. v.; Usrey, M. L.; Strano, M. S. Concomitant Length and Diameter Separation of Single-Walled Carbon Nanotubes. *J Am Chem Soc* **2004**, *126* (44), 14567–14573. https://doi.org/10.1021/ja046450z.

(9) Zhao, P.; Amani, M.; Lien, D. H.; Ahn, G. H.; Kiriya, D.; Mastandrea, J. P.; Ager, J. W.; Yablonovitch, E.; Chrzan, D. C.; Javey, A. Measuring the Edge Recombination Velocity of Monolayer Semiconductors. *Nano Lett* **2017**, *17* (9), 5356–5360. https://doi.org/10.1021/acs.nanolett.7b01770.

# 2. Theoretical Aspects

The microscopical model that we use for carbon-nanotubes is very similar to the model we have used in Ref. [1]. There, we have successfully described the experiment with aligned carbon-nanotubes that we compare with the random carbon-nanotubes in this work. However, we have made few changes in order to even better describe the experimental data which we discuss in this chapter. Furthermore, we will explain in detail how we model the randomness of the carbon-nanotubes.

## 2.1 Bandstructure

We describe the carbon-nanotubes with a number of different quasi-particles: electrons, phonons, excitons, and photons. All these quasi-particles have different dispersion relations $\epsilon_n(k)$ where $n$ is the quasi-particle-band index and $k$ is the momentum along the tube axis.

The bands we are using are exactly the same as in Ref. [1] (Fig. S5). Summarizing, we use three different excitons: the bright $E_{22}$ mode ($E_{22}$), the bright $E_{11}$ mode ($E_{11}$) and the dark singlet $E_{11}$ mode ($dE_{11}$) from Ref. [2]. We furthermore adjust the energetic position of the bands according to our absorption spectrum (Fig. 1c in Ref. [1]).

We describe the free charge carriers with four electron bands (from lowest energetic to highest): $e_1$, $e_2$, $e_3$ and $e_4$. For these bands we use the dispersion relation from a graphene tight-binding model in zone-folding approximation[3]. The energetic position of the electron bands are adjusted to be consistent with the exciton bands and the binding energies from Ref. [4].

Carbon-nanotubes have a large number of phononic bands. We do not include all of them but rather two where the first band resembles the acoustic 2nd A-branch of Fig. 3 in Ref. [5] ($ac$) and the second band is an effective Einstein-like optical phonon with an energy $\epsilon_{\text{op}} = 0.1\text{eV}$ ($op$). In addition we include a flat phonon-like band at zero energy to mimic the impurities ($imp$).

In addition we include a steep dispersion to describe photons for the laser-excitation ($h\nu$).

For more details see the supplementary material of Ref. [1].

## 2.2 Boltzmann transport equation

We calculate the quasi-particle dynamics in carbon-nanotubes with the so-called Boltzmann-transport-equation (BTE). There, the quasi-particles are described by distribution-functions $f_n(k,t)$ where $n$ is the band-index that also includes the quasi-particle type (i.e. electrons, phonons, photons, excitons), $k$ is the continuous momentum along the tube-axis

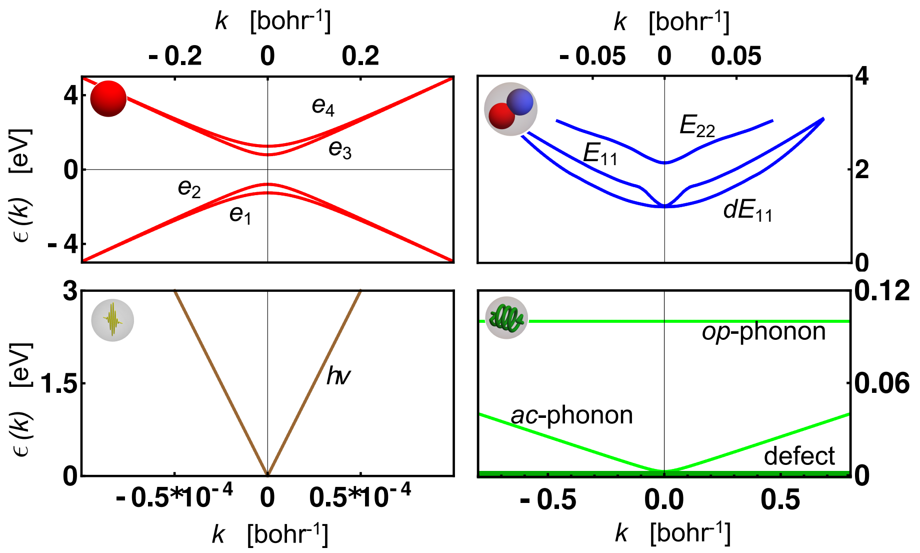


Fig. S5: The electron bands $e_{1...4}$, excitonic bands $E_{11}$, $dE_{11}$, $E_{22}$, the photon band $h\nu$ and the phonons $ac$, $op$ with an additional defect/impurity ($imp$) band. This figure was already published in Ref. [1].

and $t$ is the time. The distribution functions give the probability that a certain quasi-particle state $n, k$ is occupied at time $t$. The BTE describes the time-evolution of the distribution-functions and reads

$$\frac{\partial}{\partial t} f_n(k,t) + q_n E \frac{\partial}{\partial k} f_n(k,t) = \sum_i \left( \frac{\partial f_n(k,t)}{\partial t} \right)_{\text{col-i}} \tag{2.1}$$

where $E$ is the external electric field, $q_n$ is the quasi-particle charge and $\left( \frac{\partial f_n(k,t)}{\partial t} \right)_{\text{col-i}}$ are the collision-operators that describe the effect of scatterings between different quasi-particles. For example, the collision-operator for the process where an electron in band $e_1$ is scattered to the band $e_2$ due to the absorption of an acoustic phonon $ac$ (or the time-reversed process) is given by

$$\begin{aligned} \left( \frac{\partial f_{e_1}(k_0)}{\partial t} \right)_{e_1 + ac \leftrightarrow e_2} &= \\ = \iint_{V_{BZ}{}^2} \mathrm{d}k_1 \mathrm{d}q \; w^{\text{e-ph}}(k_0, k_1, q) \; \delta(k_1 - k_0 - q) \; \delta(\epsilon_{e_1}(k_0) - \epsilon_{e_2}(k_1) + \epsilon_{ac}(q)) \\ \times \Big[ (1 - f_{e_1}(k_0)) f_{e_2}(k_1)(1 + f_{ac}(q)) - f_{e_1}(k_0)(1 - f_{e_2}(k_1)) f_{ac}(q) \Big] \; . \end{aligned} \tag{2.2}$$

The above equation contains the distribution-functions of all the involved quasi-particles, two delta distributions that ensure energy- and momentum-conservation and the so-called scattering amplitude $w^{\text{e-ph}}$ that is proportional to the absolute square of the transition-matrix element of the initial- and final states of the scattering with the interaction-potential.

The numerically most demanding part in solving the BTE are the collision operators as they scale badly with the desired precision. Additionally, the requirements regarding particle- , energy- and momentum conservation are high in thermalization simulations. For more details on the numerical issues and the method used to actually calculate the scatterings please see Ref. [6].

| Laser-absorption | Autoionization | Electron-phonon | Exciton-phonon | Exciton impact-gen |
|---|---|---|---|---|
| $E_{22} + Imp \leftrightarrow h\nu$ | $e_1 + E_{22} \leftrightarrow e_3$ | $e_1 + ac \leftrightarrow e_1$ | $E_{11} + ac \leftrightarrow E_{11}$ | $e_2 + Imp + dE_{11} \leftrightarrow e_2$ |
| | $e_2 + E_{22} \leftrightarrow e_3$ | $e_1 + op \leftrightarrow e_1$ | $E_{11} + op \leftrightarrow E_{11}$ | $e_3 + Imp + dE_{11} \leftrightarrow e_3$ |
| | $e_2 + E_{22} \leftrightarrow e_4$ | $e_2 + ac \leftrightarrow e_2$ | $dE_{11} + ac \leftrightarrow dE_{11}$ | $e_3 + Imp + dE_{11} \leftrightarrow e_4$ |
| | $e_1 + E_{11} \leftrightarrow e_3$ | $e_2 + op \leftrightarrow e_2$ | $dE_{11} + op \leftrightarrow dE_{11}$ | $e_1 + Imp + dE_{11} \leftrightarrow e_2$ |
| | $e_2 + E_{11} \leftrightarrow e_3$ | $e_3 + ac \leftrightarrow e_3$ | $E_{22} + ac \leftrightarrow E_{22}$ | $e_2 + Imp \leftrightarrow dE_{11} + e_2$ |
| | $e_2 + E_{11} \leftrightarrow e_4$ | $e_3 + op \leftrightarrow e_3$ | $E_{22} + op \leftrightarrow E_{22}$ | $e_3 + Imp \leftrightarrow dE_{11} + e_3$ |
| | $e_1 + dE_{11} \leftrightarrow e_3$ | $e_4 + ac \leftrightarrow e_4$ | $E_{11} + ac \leftrightarrow dE_{11}$ | $e_4 + Imp \leftrightarrow dE_{11} + e_3$ |
| | $e_2 + dE_{11} \leftrightarrow e_3$ | $e_4 + op \leftrightarrow e_4$ | $E_{11} + op \leftrightarrow dE_{11}$ | $e_2 + Imp \leftrightarrow dE_{11} + e_1$ |
| | $e_2 + dE_{11} \leftrightarrow e_4$ | $e_1 + ac \leftrightarrow e_2$ | $dE_{11} + ac \leftrightarrow E_{11}$ | |
| | | $e_2 + ac \leftrightarrow e_1$ | $dE_{11} + op \leftrightarrow E_{11}$ | |
| | | $e_1 + op \leftrightarrow e_2$ | $E_{22} + ac \leftrightarrow E_{11}$ | |
| | | $e_2 + op \leftrightarrow e_1$ | $E_{22} + op \leftrightarrow E_{11}$ | |
| | | $e_3 + ac \leftrightarrow e_4$ | $E_{11} + ac \leftrightarrow E_{22}$ | |
| | | $e_4 + ac \leftrightarrow e_3$ | $E_{11} + op \leftrightarrow E_{22}$ | |
| | | $e_3 + op \leftrightarrow e_4$ | $E_{22} + ac \leftrightarrow dE_{11}$ | |
| | | $e_4 + op \leftrightarrow e_3$ | $E_{22} + op \leftrightarrow dE_{11}$ | |
| | | | $dE_{11} + ac \leftrightarrow E_{22}$ | |
| | | | $dE_{11} + op \leftrightarrow E_{22}$ | |

Table 2.1: All 52 scattering processes grouped into five families as described in the text. This table was already published in the supplementary material of Ref. [1]

The transport part of the BTE that describes the collision-less motion of quasi-particles when an external field is present (i.e. the left-hand side of Eq. (2.1)) is calculated numerically with a finite-element Discontinuous Galerkin scheme.

## 2.3 Scattering processes

The scattering-amplitudes $w^{\text{col.-i}}$ depend, at least in principle, on all the momenta of the quasi-particles involved in the scattering process. In this work we make the approximation of momentum-independent scattering amplitudes.

In total, we include 52 scattering processes where each has its individual scattering amplitude (Table 2.1). As it is difficult if not impossible to deduce each individual scattering amplitude from experiments we logically group the scatterings into five groups with the same scattering amplitude for all these processes (Table 2.1). In the following we explain the different scattering groups and the corresponding scattering amplitudes.

### 2.3.1 Laser

In the experiments, the carbon-nanotubes are irradiated with a laser that hits the $E_{22}$ resonance. In principle, the laser excitation corresponds to a two-leg process ($h\nu \to E_{22}$) which produces excitons that are all located at the Gamma-point. However, directly after the excitation, the produced excitons scatter with acoustic phonons and impurities and are therefore redistributed to other momenta at approximately the same energy. As we are not interested in the exact dynamics during the laser absorption we instead treat the laser excitation as a three-leg scattering process, i.e. impurity-assisted excitation. This can be seen as a combination of laser excitation with direct subsequent impurity scattering. We model the laser with a constant photon-population $f_{h\nu}(k) =$

$\alpha \left( \mathrm{Exp}[-\frac{(k-k_m)^2}{2\sigma^2}] + \mathrm{Exp}[-\frac{(k+k_m)^2}{2\sigma^2}] \right)$ with $k_m = 3.5667 \times 10^{-5}$ bohr$^{-1}$, $\alpha = 10^{-3}$ a.u. and $\sigma = 1.6667 \times 10^{-6}$ bohr$^{-1}$. We apply a Gaussian time profile to the scattering-amplitude $w^{E_{22}\,+\,Imp\,\leftrightarrow\,h\nu}(t) = \mathrm{Exp}[-\frac{(t-t_c)^2}{2{\sigma_t}^2}]$ with the center-time $t_c = 0.5ps$ and the temporal broadening $\sigma_t = 0.1$ps. These parameters reproduce the laser used in the experiments, i.e. a laser with center-frequency of $\hbar\omega = 2.14$eV (in resonance with $E_{22}$) and an energetic broadening of $\sigma_{en} = 0.1$eV.

The above laser-parameters correspond to an excitation that produces excitons in the small occupation regime. We use the same laser for the aligned as well as the random carbon-nanotubes.

#### 2.3.2 Exciton autoionization

As found and discussed in detail in Ref. [1] excitons with sufficient energy may dissociate into free carriers. We choose an exciton autoionization scattering amplitude $w = 9.3 \times 10^{-6}$ a.u. that corresponds to an autoionization lifetime of 0.1ps.

This is the same value used in Ref. [1] where a more detailed discussion can be found in its supplementary material. We use the same scattering amplitude for the aligned as well as the random carbon-nanotubes.

#### 2.3.3 Exciton-phonon scattering

Several experiments suggest that the decay of $E_{22}$ excitons to the bottom of the bright $E_{11}$ takes place in few hundred femto-seconds[7, 8, 9]. The only mechanism to dissipate energy in the low occupation regime is through optical phonon emission. We use an optical phonon scattering amplitude of $w^{op} = 60.5 \times 10^{-6}$ a.u. which corresponds to an emission of an optical phonon every 40fs on average. It then takes about 400fs for an $E_{22}$ exciton to decay to the bottom of the $E_{11}$ and $dE_{11}$ bands. The acoustic phonons have little effect on the dynamics as they mainly transfer momentum and dissipate only very little energy. Therefore, in order to reduce numerical cost we use a smaller scattering amplitude of $w^{ac} = 4.84 \times 10^{-6}$ a.u..

The above scattering amplitudes are the same as in Ref. [1] and we use these parameters for the aligned as well as for the random carbon-nanotubes.

#### 2.3.4 Electron-phonon and electron-impurity scattering

In Ref. [1] we have taken the value of 50fs suggested by Ref. [10] for the most effective phonon scattering as the reference value to determine the scattering-amplitude. However, this was only a lower boundary for the scattering time. Within our new set of parameters we find that we have an overall better agreement with the experimental data with a scattering amplitude that is one fifth of the value used in Ref. [1], i.e. the new value of the scattering amplitude is $w^{e-ph} = 0.968 \times 10^{-6}$ a.u..

For the aligned carbon-nanotubes we use this $w^{e-ph}$ scattering amplitude for acoustic- as well as for optical-phonon scattering. One effect of the randomness in the random carbon-nanotube sample is expected to be an increased impurity-like scattering due to the multiple tube distortions coming from intersecting tubes. In the dynamics, impurity scattering has the effect of momentum-dissipation and is, a-priori, indistinguishable from scattering with acoustic phonons if we consider only one temperature. Therefore we use an increased acoustic phonon-scattering of $w^{e-ac} = 10 \times w^{e-ph}$ for the random CNTs.

The optical-phonon scattering strength we leave the same as in the aligned tubes, i.e. $w^{e-ac} = w^{e-ph}$.

### 2.3.5 Exciton-impact generation

One of the findings in our previous work Ref. [1] was that the super-linear dependence of the photo-current on the applied bias originates from exciton-impact generation. In accordance with Ref.[11] we found that the direct process is not possible except at very high energies due to simultaneous momentum- and energy-conservation. We have modeled the impact generation process as an impurity-assisted process. The impurity breaks the momentum-conservation which makes the process possible at reasonable energies. Instead of an impurity, also a phonon or the next higher electronic band could act as a source for the additional momentum.

In Ref. [11] the life-time of high-energetic electrons due to impact excitation is calculated. Most of the given values lie in the range 0.1-1ps. In our previous work we have chosen the scattering rate in accordance with a life-time of 1ps which gave the correct ratio between the linear and the super-linear part in the photo-current. Here, we use a scattering-amplitude that is 5-times larger than in Ref.[1], i.e. $w^{imp} = 24.2 \times 10^{-5}$ a.u.. This scattering-amplitude corresponds to an electron life-time due to this process of about 0.2ps. We find that this larger value is needed to maintain the correct ratio between the linear and super-linear part in the photo-current when the new scattering-amplitudes for electron-phonon scattering and the increased extraction rate presented here, are used.

## 2.4 Electron-electron scattering

As in Ref. [1] we exclude electron-electron scatterings as they scale quadratically with the occupation and we perform simulations only in the low occupation regime.

## 2.5 Carrier extraction from CNTs

In our model used in Ref. [1] we have assumed that the main mechanism behind the switching-off of the electric current is the extraction of carriers into the metallic leads. As we do not describe the dynamics in the carbon-nanotubes with the full-fledged momentum- and real-space-dependent Boltzmann-transport-equation, but rather with a version that only takes the momentum into account, we need to model this extraction of the carriers. This is achieved by a decay term with the shape

$$\left(\frac{\partial f_n(k)}{\partial t}\right)_{\text{extr.}} = -\frac{1}{\tau_{\text{extr.}}}\left(f_n(k) - f_{n-eq.}(k)\right) \quad n \in \{e_1, \ldots, e_4\} \tag{2.3}$$

where the extraction time $\tau_{\text{extr.}}$ is the characteristic time the electron stays in the carbon-nanotubes and $f_{n-eq.}(\cdot)$ is the equilibrium distribution of band $n$, i.e. the initial distribution. In Ref. [1] we have used the length of the sample to estimate the extraction time. We have come to the conclusion that this underestimated the electron extraction-rate, which is also supported by the fact that the second half-wave of the terahertz radiation was too small in magnitude. As an alternative decay mechanism we have suggested recombination once the carrier reaches the edge of the carbon-nanotube. We use this scenario to estimate the extraction time here.

For that purpose we decrease the extraction time by a factor of 5 relative to the value used in Ref. [1] which leads to a new extraction-time of $\tau_{\text{extr.}} \approx 1\text{ps}$.

The maximum velocity of the carriers in CNTs is $v_{\max} \approx 1\mu\text{m/ps}$ and the average length of an single tube is $l_{\text{CNT}} \approx 420\text{nm}$[12]. Assuming, that the average carrier starts from the middle of the tube, the average velocity of a carrier with an extraction time of 1ps is $\bar{v} = (l_{\text{CNT}}/2)/\tau_{\text{extr.}} = 0.21\mu\text{m/ps} \approx v_{\max}/5$ which is a reasonable value for the average velocity.

## 2.6 Initial configuration

As initial configuration we use Fermi-Dirac distributions with a chemical potential $\mu = 0$ and inverse temperature $\beta = 35\text{eV}^{-1}$ for the electronic bands $e_{1\ldots4}$. The temperature that corresponds to this $\beta$ is $T \approx 330\text{K}$. We have chosen a temperature higher than room temperature to partially take into account the heating of the sample.

The initial distribution-functions of the acoustic- and optical-phonon bands are Bose-Einstein distributions with $\mu = 0$ and the $\beta = 35\text{eV}^{-1}$. As we approximate the phononic system with two bands only, we keep the phononic distributions fixed to their initial value throughout the whole simulation to model the higher heat capacity of the full phononic system.

The impurity distribution is kept to zero throughout the whole simulation. It can be shown that a phononic scattering with a distribution function that equals zero produces the same statistical factor as impurity scattering.

## 2.7 Electric field in aligned- and non-aligned-CNTs

The electric field along the tube axis can be calculated from the applied bias $U$. The length of the CNT sample between the metallic leads is $l = 6\mu\text{m}$ and we use a dielectric constant of $\epsilon = 2$. With this, the electric field in the aligned carbon-nanotubes can be calculated as

$$E_{\text{align}} = \frac{U}{l\epsilon} \ . \tag{2.4}$$

However, in the non-aligned carbon-nanotubes the electric field along the tube axis is reduced by a factor $\cos\varphi$ where $\varphi$ is the angle between the tube axis and the direction of the electric field. In case of the random carbon-nanotube sample, this angle is a random number in the interval $\varphi \in [-{}^{\pi}/_{2}, {}^{\pi}/_{2})$ for each tube. Note, that each angle has the same probability. The expectation value of the electric field is then

$$E_{\text{rand}} = \frac{1}{\pi}\int_{-\pi/2}^{\pi/2} \mathrm{d}\varphi \frac{U}{l\epsilon}\cos\varphi = \frac{2}{\pi}\frac{U}{l\epsilon} \ , \tag{2.5}$$

i.e. by a factor ${}^{2}/_{\pi}$ smaller than in a comparable aligned sample.

## 2.8 Calculation of THz radiation

It can be shown that the electric field of an emitted electromagnetic wave is proportional to the time-derivative of the electric current in the sample, i.e.

$$E_{THz}(t) \propto \frac{\partial I(t)}{\partial t} \ . \tag{2.6}$$

Within the Boltzmann framework the electric current is calculated from the distribution-functions as

$$I(t) = \frac{1}{2\pi} \sum_{n=e_1}^{e_4} \int \mathrm{d}k \frac{\partial \epsilon_n(k)}{\partial k} q_n f_n(k,t) \ . \tag{2.7}$$

## 2.9 Calculation of the photocurrent

In our model we assume, that the long-time current is mainly dominated by low-energetic $E_{11}$-excitons which slowly thermally dissociate. As a result, there is a constant source of free carriers which are then accelerated in the electric field and produce a current. We do not simulate this long-time current as this would have been beyond the numerical capabilities of the current implementation of the code.

We rather estimate the current as

$$I_{\mathrm{photo}} \propto n_{11} \times U \ , \tag{2.8}$$

where $n_{11}$ is the total $E_{11}$-exciton number after the initial, quick dynamics is over and $U$ is the applied voltage. This is reasonable as the long-time current will be proportional to the number of free carriers (which itself is proportional to the exciton-number) and the voltage that drives the current.

In general, the exciton number $n_{11}$ depends on the applied bias $U$ in a non-linear fashion. Therefore, in case of random CNTs we would have to take the average among all differently oriented CNTs to determine the expectation value of the photocurrent. However, we find that in our model the final exciton number does not depend on the voltage in case of the random CNTs. Therefore, the expectation value only changes the voltage by a factor of $^2/_\pi$ as explained in section 2.7. Since Eq. (2.8) is only a proportionality relation we can use it in the same way as for aligned CNTs.

# Bibliography


[1] Bagsican, F. R. G. et al. Terahertz excitonics in carbon nanotubes: Exciton autoionization and multiplication. Nano Letters **20**, 3098–3105 (2020). URL https://doi.org/10.1021/acs.nanolett.9b05082. PMID: 32227963, https://doi.org/10.1021/acs.nanolett.9b05082.

[2] Dresselhaus, M. S., Dresselhaus, G., Saito, R. & Jorio, A. Exciton photophysics of carbon nanotubes. Annual Review of Physical Chemistry **58**, 719–747 (2007). URL https://doi.org/10.1146/annurev.physchem.58.032806.104628. PMID: 17201684, https://doi.org/10.1146/annurev.physchem.58.032806.104628.

[3] Charlier, J.-C., Blase, X. & Roche, S. Electronic and transport properties of nanotubes. Rev. Mod. Phys. **79**, 677–732 (2007). URL https://link.aps.org/doi/10.1103/RevModPhys.79.677.

[4] Maultzsch, J. et al. Exciton binding energies in carbon nanotubes from two-photon photoluminescence. Phys. Rev. B **72**, 241402 (2005). URL https://link.aps.org/doi/10.1103/PhysRevB.72.241402.

[5] Lim, Y.-S. et al. Ultrafast generation of fundamental and multiple-order phonon excitations in highly enriched (6,5) single-wall carbon nanotubes. Nano Letters **14**, 1426–1432 (2014). URL https://doi.org/10.1021/nl404536b. PMID: 24527806, https://doi.org/10.1021/nl404536b.

[6] Wais, M., Held, K. & Battiato, M. Numerical solver for the time-dependent far-from-equilibrium boltzmann equation. arXiv:2004.02683 (2020).

[7] Reich, S., Dworzak, M., Hoffmann, A., Thomsen, C. & Strano, M. S. Excited-state carrier lifetime in single-walled carbon nanotubes. Phys. Rev. B **71**, 033402 (2005).

[8] Lauret, J.-S. et al. Ultrafast carrier dynamics in single-wall carbon nanotubes. Phys. Rev. Lett. **90**, 057404.

[9] Manzoni, C. et al. Intersubband exciton relaxation dynamics in single-walled carbon nanotubes. Phys. Rev. Lett. **94**, 207401 (2005).

[10] Park, J.-Y. et al. Electron-phonon scattering in metallic single-walled carbon nanotubes. Nano Letters **4**, 517–520 (2004).

[11] Baer, R. & Rabani, E. Can impact excitation explain efficient carrier multiplication in carbon nanotube photodiodes? Nano Letters **10**, 3277–3282 (2010).

[12] He, X. et al. Wafer-scale monodomain films of spontaneously aligned single-walled carbon nanotubes. Nature Nanotechnology **11**, 633–638 (2016). URL https://doi.org/10.1038/nnano.2016.44.
